# The Effects of Cognitive Reappraisal and Expressive Suppression on Memory of Emotional Pictures

**DOI:** 10.3389/fpsyg.2017.01921

**Published:** 2017-11-01

**Authors:** Yan Mei Wang, Jie Chen, Ben Yue Han

**Affiliations:** The School of Psychology and Cognitive Science, East China Normal University, Shanghai, China

**Keywords:** emotion regulation, cognitive reappraisal, expressive suppression, emotional pictures, memory

## Abstract

In the field of emotion research, the influence of emotion regulation strategies on memory with emotional materials has been widely discussed in recent years. However, existing studies have focused exclusively on regulating negative emotion but not positive emotion. Therefore, in the present study, we investigated the influence of emotion regulation strategies for positive emotion on memory. One hundred and twenty college students were selected as participants. Emotional pictures (positive, negative and neutral) were selected from Chinese Affective Picture System (CAPS) as experimental materials. We employed a mixed, 4 (emotion regulation strategies: cognitive up-regulation, cognitive down-regulation, expressive suppression, passive viewing) × 3 (emotional pictures: positive, neutral, negative) experimental design. We investigated the influences of different emotion regulation strategies on memory performance, using free recall and recognition tasks with pictures varying in emotional content. The results showed that recognition and free recall memory performance of the cognitive reappraisal groups (up-regulation and down-regulation) were both better than that of the passive viewing group for all emotional pictures. No significant differences were reported in the two kinds of memory scores between the expressive suppression and passive viewing groups. The results also showed that the memory performance with the emotional pictures differed according to the form of memory test. For the recognition test, participants performed better with positive images than with neutral images. Free recall scores with negative images were higher than those with neutral images. These results suggest that both cognitive reappraisal regulation strategies (up-regulation and down-regulation) promoted explicit memories of the emotional content of stimuli, and the form of memory test influenced performance with emotional pictures.

## Introduction

In daily life, the various emotion-triggering events (ETE) (Sheppes and Meiran, 2007) we encounter evoke different emotional experiences. Emotions play a crucial role in survival because they remind us to seek advantages and avoid disadvantages. However, highly intense emotions are not always adaptive. Successful management of our emotions is associated with positive outcomes, including an enhanced sense of self-efficacy and improved psychological well-being (Eisenberg et al., 2000). Emotion regulation refers to the processes by which we influence which emotions we experience, when we experience them, and how we experience and express them (Gross, 1998b). Different emotion regulation strategies can produce different effects on personal experiences, cognition, social behaviors, and well-being (Gross, 2002; Phillips et al., 2006; Rubin et al., 2009). The effects of various emotion regulation strategies on cognition and memory have received considerable attention from researchers (Dillon et al., 2007; Hayes et al., 2010; Knight and Ponzio, 2013).

Researchers have proposed five kinds of emotion regulation strategies: situation selection, situation modification, attention deployment, cognitive reappraisal, and expressive suppression (Gross, 1998a,b, 2002). Studies have shown that cognitive reappraisal and expressive suppression were the most commonly used and most effective of these (Dillon et al., 2007; Hayes et al., 2010; Knight and Ponzio, 2013). Cognitive reappraisal is an antecedent-focused emotion regulation strategy, occurring in the early stages of the experience of the emotion. This strategy changes emotional experiences successfully by modulating cognitive processes, involving the re-interpretation of emotional events (Gross, 1998a). In previous studies investigating the impact of cognitive reappraisal on memory, most researchers found that cognitive reappraisal enhanced memories of emotional stimuli compared with passive viewing (Richards et al., 2003; Dillon et al., 2007; Hayes et al., 2010; Kim and Hamann, 2012), but some studies also found that cognitive reappraisal did not enhance the memory of an emotional stimulus relative to passive viewing condition (Richards and Gross, 1999, 2000; Erk et al., 2010).

Expressive suppression is a response-focused emotion regulation strategy that occurs in the late stage of the emotion, by suppressing emotional activities (such as facial expression) which will occur or are occurring to regulate emotion experience. Most of the previous studies have shown that expressive suppression could impair the memory of emotional events relative to a passive viewing condition (Richards and Gross, 2000; Bonanno et al., 2004; Dillon et al., 2007; Hayes et al., 2010; Binder et al., 2012).

Previous studies have shown that cognitive reappraisal had a positive effect on the memory of emotional materials (Dillon et al., 2007; Hayes et al., 2010; Kim and Hamann, 2012). For example, Hayes et al. (2010) presented negative pictures to participants and asked them to adopt a cognitive reappraisal emotion regulation strategy. Two weeks later, participants were tested on their recognition performance. The memory scores of the cognitive reappraisal group were better than that of the passive viewing group.

Researchers have further divided cognitive reappraisal into up-regulation and down-regulation of emotion to investigate their memory effects. Dillon et al. (2007) randomly assigned participants into cognitive up-regulation, down-regulation, expressive suppression, and passive viewing groups. Participants were asked to view negative pictures, then had to recall them freely. Compared with passive viewing, both the up-regulation and down-regulation conditions enhanced the memory of negative pictures, but the expressive suppression condition impaired the memory of negative pictures.

In a recent study, participants were asked to regulate their emotions by using cognitive up-regulation or down-regulation strategies while viewing negative images (Kim and Hamann, 2012). A recognition test after 2 weeks revealed that performance of male participants was significantly higher in cognitive up-regulation and cognitive down-regulation trials than in passive viewing trials. However, in a free recall test, the results failed to show improving effect of cognitive reappraisal strategy for negative images.

In a study by Knight and Ponzio (2013), the differential effects of cognitive reappraisal strategies of up-regulation and down-regulation were compared. In this study, positive pictures were added to emotional pictures. These researchers found that, in a free recall test of emotional pictures, relative to a control group cognitive up-regulation promoted memory performance, while cognitive down-regulation impaired memory performance for emotional pictures. During another strategy test, the researchers examined memory performance for the type of emotion regulation strategies participants used to evaluate each picture. Participants were instructed to correctly match each picture to the strategy they had used while viewing it. In this test, the memory performance for strategy–picture pairings of both the up-regulation and down-regulation groups was significantly better than that of a control group.

Some researchers (for example, Binder et al., 2012) have investigated the effect of expressive suppression strategies on memory performance with emotional materials, always finding that expressive suppression damaged memory performance. Bonanno et al. (2004) examined the effects of expressive suppression on the memory of emotional pictures. They also performed further subdivisions of expressive suppression: one group of participants were asked to enhance facial expressions, while participants in another group were asked to suppress facial expressions, and a third group were just asked to view passively. The researchers then examined participants’ memories of the details of the slides. The results showed that compared with the control group, both enhancing and suppressing expressions resulted in impaired memory performance.

Some researchers have reported that, compared with expressive suppression and passive viewing, cognitive reappraisal could enhance memory of emotional materials; however, other studies have indicated otherwise (Richards and Gross, 1999, 2000; Erk et al., 2010). After dividing cognitive reappraisal into up-regulation and down-regulation of emotion, the effects of cognitive reappraisal and expressive suppression on memorizing emotional materials are unclear. Indeed, only a handful of studies have examined the differential effects of these three emotion regulation strategies (expressive suppression, up-regulation of emotion, down-regulation of emotion) on explicit memory using both recognition and free recall tasks. In addition, a limited number of studies have examined the effects of voluntary regulation of positive emotions on memory. However, it is essential to regulate both negative and positive emotions in daily life. To further explore the effects of emotion regulation strategies on the memory of emotional photos, the present study manipulated three emotion regulation strategies: up-regulation of emotion, down-regulation of emotion, and expressive suppression. Negative pictures, neutral pictures, and positive ones, were used as stimulating materials. We also investigated the performance of two kinds of explicit memory, namely free recall and recognition. We hypothesized that cognitive reappraisal strategies would reinforce explicit memory. We also predicted that participants would recall and recognize more emotional (unpleasant and pleasant) pictures than neutral ones.

## Materials and Methods

### Participants

One hundred and twenty undergraduate students (80 females, 40 males, aged 18–23 years old [*M ± SD* = 20.02 ± 0.43 years]) participated. The study was approved by the local ethics committee, with all participants providing written informed consent.

### Stimulus

As a cultural bias for the International Affective Picture System (IAPS) has been reported in Chinese participants (Huang and Luo, 2004; Liu et al., 2009; Gong and Wang, 2016), the 60 pictures (20 negative, 20 neutral and 20 positive) we used in the current study were selected from the native Chinese Affective Picture System (Chen et al., 2008; Li et al., 2008; Yuan et al., 2008). Normative mean valences (*SD*) (1 = very unpleasant, 9 = very pleasant) and arousal (1 = very calming, 9 = very excited) ratings were 1.89 (0.43) and 5.63 (0.16) for negative pictures, 7.03 (0.27) and 5.64 (0.25) for positive pictures and 5.38 (0.23) and 4.10 (0.20) for neutral pictures. The CAPS arousal ratings for positive and negative pictures did not differ significantly, *t*(58) = 0.36, *p* > 0.05. Positive and negative images differed in valence, *t*(58) = 53.55, *p* < 0.001.

Thirty pictures (10 negative, 10 neutral, and 10 positive) were presented at encoding procedure, and the others as novel stimuli in the recognition test. Pictures in the encoding procedure and recognition tests were counterbalanced across participants. Participants were randomly assigned to each of four groups: passive viewing, expressive suppression, cognitive up-regulation, and cognitive down-regulation. Each group had 30 participants.

### Encoding Procedure

**FIGURE 1:**
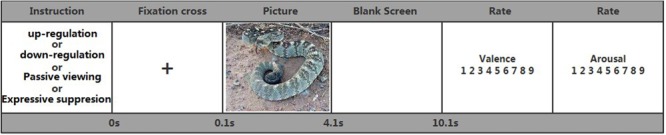
Time course of trials.

After participants gave informed consent, instructions were provided. These informed participants which emotion regulation strategy they should use to regulate their emotion. Each trial (see **Figure 1**) began with a fixation cross for 100 ms. The fixation cross was followed by a picture that was either positive, negative or neutral (4000 ms). The picture was then replaced with a blank screen (6000 ms). Immediately following the blank screen, participants rated how strongly they experienced negative or positive emotion on a computerized scale ranging from 1 (very negative) to 9 (very positive), and how intense their experience was on a scale ranging from 1 (very calm) to 9 (very intense). Valence and intensity ratings occurred at the end of each trial. Participants were given opportunities to practice the strategies with pictures from each valence category.

Detailed instructions of four different strategies were provided. For the “passive viewing” condition, participants were instructed to let their reactions to the pictures unfold naturally. For the “up-regulation” condition, participants were instructed to intensify any emotions they experienced by increasing the personal relevance of the picture. Participants were told to imagine himself or herself or a loved one as the central figure in the scene. For the “down-regulation” condition, participants were instructed to diminish their emotional reactions by decreasing the personal relevance of the picture. Participants were told to view the images from the perspective of a detached observer, the third-person perspective. For the “expressive suppression” condition, participants were instructed to try their best not to let those feelings show. They were asked to try to behave in such a way that “a person watching you would not know you are feeling anything at all.”

### Recognition Test

After the encoding procedure, participants were asked to complete a recognition test. Sixty pictures were randomly presented. Thirty of them had appeared in the encoding trials. Participants were told to judge whether the picture on the screen had previously appeared, and to press “F” if the answer was yes, and press “J” if the answer was no. Participants also had to ensure that the speed and accuracy of reaction.

### Free Recall

Following the recognition test, there was a free recall test. Participants were instructed to provide a written description for as many pictures as they could recall from the encoding phase, and were asked to provide enough detail so that an outsider could identify each picture and could differentiate it from similar pictures. They were told to avoid using abstract words, such as beautiful, nausea, and so forth. Free recall data were scored according to the number of pictures’ features that participants reported. Each picture had one crucial feature and 1–2 detailed features; these features were enough to allow all raters to identify and classify the corresponding pictures as remembered. Zero and 1 codes represented a complete retrieval failure and a retrieval success, respectively. Participants were also told that they could list the pictures in any order and that they could have as much time as they needed. Recall was scored by three students who majored in psychology. Inter-rater reliability was very high (Cronbach’s α = 0.99).

## Results

### Valence and Arousal Ratings

Valence and arousal ratings were subjected to a repeated measures ANOVA with strategy (up-regulation, down-regulation, expressive suppression, passive viewing) as the between-subjects factor and emotional picture (negative, neutral, and positive) as the within-subjects factor.

For the valence ratings (see **Table 1**), the results showed a significant emotional picture × strategy interaction, *F*(6,72) = 2.106, *p* < 0.05. For positive and neutral pictures, emotional experience was least pleasant for down-regulation, intermediate for expressive suppression and passive viewing, and most pleasant for up-regulation. In contrast, for negative pictures, emotional experience was most pleasant for down-regulation, intermediate for expressive suppression and passive viewing, and least pleasant for up-regulation. There was a significant main effect of emotional pictures, *F*(2,36) = 445.80, *p* < 0.001. Valence ratings were lowest for trials featuring negative pictures, intermediate for trials featuring neutral pictures, and highest for trials featuring positive pictures (all *p*s < 0.05). The results also showed a significant main effect of strategies, *F*(3,36) = 13.48, *p* < 0.001, with the order of valence ratings from low to high being down-regulation, passive viewing and expressive suppression, up-regulation (all *p*s < 0.05).

**Table 1:** Mean self-reported valence and arousal by picture valence and strategy.

	Positive	Neutral	Negative
**Valence rating**			
Up-regulation	7.23 (0.21)	5.46 (0.15)	2.26 (0.47)
Down-regulation	6.59 (0.15)	5.26 (0.15)	3.94 (0.44)
Suppression	6.81 (0.22)	5.19 (0.13)	2.39 (0.45)
Passive viewing	7.02 (0.20)	5.46 (0.17)	2.56 (0.47)
**Arousal rating**			
Up-regulation	6.78 (0.39)	3.81 (0.51)	6.89 (0.42)
Down-regulation	3.67 (0.35)	2.62 (0.26)	5.44 (0.70)
Suppression	4.57 (0.45)	2.72 (0.47)	6.52 (0.48)
Passive viewing	5.65 (0.27)	3.10 (0.39)	6.26 (0.44)

*Mean and standard deviation.*

For the arousal ratings of positive and neutral pictures (see **Table 1**), the results showed a significant emotional picture × strategy interaction, *F*(6,72) = 16.23, *p* < 0.001, $\eta_{p}^{2}$ = 0.56. Compared to the passive viewing condition (2.80 ± 1.44), arousal ratings were higher (*p* < 0.05) in the up-regulation condition, and lower (*p* < 0.05) in the down-regulation condition, regardless of the emotional content of the pictures. Arousal ratings in the passive viewing condition (4.37 ± 1.35) were significantly higher than in the expressive suppression condition (3.65 ± 1.05, *p* < 0.001) in the trials featuring positive and neutral pictures. However, for negative pictures, there was no difference in arousal ratings between the passive viewing (6.26 ± 0.44) and expressive suppression (6.52 ± 0.48) conditions. In summary, cognitive reappraisal was successful at both increasing and decreasing emotion, regardless of the pictures. The expressive suppression strategy reduced arousal ratings for positive and neutral pictures, but not for negative pictures.

There was a significant main effect for emotional picture [*F*(2,36) = 622.40, *p* < 0.001, $\eta_{p}^{2}$= 0.95] and a significant main effect for strategy [*F*(3,36) = 80.66, *p* < 0.001, $\eta_{p}^{2}$= 0.87]. Participants’ arousal ratings were lowest in trials featuring neutral pictures (3.06 ± 0.62, *p* < 0.001), intermediate in trials featuring positive pictures (5.17 ± 1.12, *p* < 0.001), and highest in trials featuring negative pictures (6.28 ± 0.74, *p* < 0.001). Participants reported higher arousal in cognitive up-regulation trials, and lower arousal in cognitive down-regulation trials and expressive suppression trials, relative to passive viewing trials.

### Recognition Performance

The two-way mixed ANOVA for recognition reaction times was performed with emotional picture (negative, neutral, positive) as a within-subjects factor and strategy (down-regulation, up-regulation, expressive suppression, passive viewing) as a between-subjects factor. Recognition performance for emotional pictures on different strategies group is summarized in **Table 2**.

**Table 2:** Mean recognition RT by picture valence and strategy.

	Positive	Neutral	Negative
Up-regulation	722.00 (71.43)	768.89 (61.39)	745.78 (73.37)
Down-regulation	714.64 (64.05)	772.39 (73.34)	772.67 (79.63)
Suppression	812.00 (93.97)	852.92 (166.15)	845.48 (132.78)
Passive viewing	765.44 (90.67)	812.70 (100.55)	841.89 (108.07)

*Mean and standard deviation.*

**FIGURE 2:**
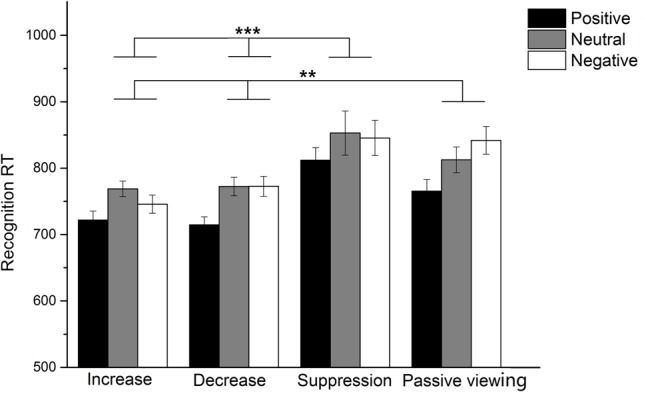
Recognition reaction times for each emotion image type in all emotion regulation strategy groups. ^∗∗^*p* < 0.01, ^∗∗∗^*p* < 0.001.

The results (see **Figure 2**) showed that the emotional picture × strategy interaction effect was not significant, *F*(6,208) = 0.95, *p* = 0.46, $\eta_{p}^{2}$= 0.03. They also revealed a significant main effect of emotional picture, *F*(2,104) = 15.80, *p* < 0.001, $\eta_{p}^{2}$= 0.13. Planned comparisons indicated that recognition reaction times were shortest for positive pictures (*M* ±*SD* = 750.89 ± 88.34, *p* < 0.001). There was no significant difference in reaction times between negative pictures (*M* ±*SD* = 799.86 ± 108.07) and neutral pictures (*M* ±*SD* = 800.38 ± 109.57). In addition, there was also a significant main effect of strategy, *F*(3,104) = 8.74, *p* < 0.001, $\eta_{p}^{2}$= 0.20. Follow-up tests revealed that recognition reaction times were shortest for pictures presented in up-regulation trials (*M* ±*SD* = 745.56 ± 70.77) and down-regulation trials (*M* ±*SD* = 753.24 ± 76.81), intermediate for pictures presented in passive viewing trials (*M* ±*SD* = 806.68 ± 133.63), and longest for pictures presented in expressive suppression trials (*M* ±*SD* = 836.80 ± 104.05; see **Figure 2**).

### Free Recall

Free recall scores were calculated by emotional picture and strategy, and were entered into a repeated-measures ANOVA with emotional picture (negative, neutral, positive) as the within-subjects factor and strategy (down-regulation, up-regulation, expressive suppression, passive viewing) as the between-subjects factor. Free recall performance for emotional pictures on different strategies group is summarized in **Table 3**.

**Table 3:** Mean score of free recall by picture valence and strategy.

	Positive	Neutral	Negative
Up-regulation	4.41 (1.02)	3.27 (1.03)	5.69 (1.71)
Down-regulation	4.93 (1.60)	3.31 (1.51)	6.75 (1.28)
Suppression	3.84 (1.12)	2.36 (1.47)	4.81 (1.47)
Passive viewing	3.87 (1.49)	2.45 (1.00)	5.39 (1.62)

*Mean and standard deviation.*

**FIGURE 3:**
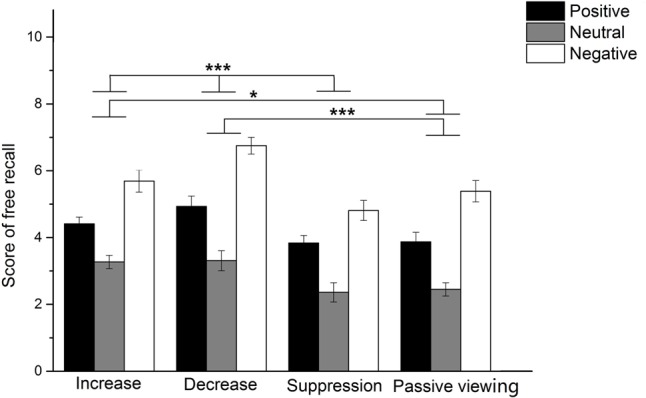
Scores on free recall task for each emotion image type in all emotion regulation strategy groups. ^∗^*p* < 0.05, ^∗∗∗^*p* < 0.001.

The results (see **Figure 3**) showed that the emotional picture × strategy interaction effect was not significant, *F*(6,200) = 0.85, *p* = 0.54. The results revealed a significant main effect of emotional picture, *F*(2,100) = 100.37, *p* < 0.001, $\eta_{p}^{2}$= 0.50. Recall accuracy was significantly higher for negative pictures (*M* ±*SD* = 5.66 ± 1.66) than for positive pictures (*M* ±*SD* = 4.26 ± 1.39). Recall performance was the lowest for neutral pictures (*M* ±*SD* = 2.85 ± 1.34). In addition, the results also showed a significant main effect of strategy, *F*(3,100) = 16.89, *p* < 0.001, $\eta_{p}^{2}$= 0.34. Free recall score was highest for pictures presented in up-regulation trials (*M* ±*SD* = 4.46 ± 1.62) and down-regulation trials (*M* ±*SD* = 5.00 ± 2.02), and there was no significant difference in free recall performance between them (*p* > 0.05). Recall was better for pictures presented in up-regulation trials than in passive viewing trials (*M* ±*SD* = 3.90 ± 1.83) and expressive suppression trials (*M* ±*SD* = 3.67 ± 1.69). There was no significant difference in free recall scores between passive viewing trials and expressive suppression trials (*p* > 0.05; see **Figure 3**).

## Discussion

The present study aimed to explore the effects of four different emotion regulation strategies (cognitive up-regulation, cognitive down-regulation, expressive suppression, and passive viewing) on explicit memory of emotional pictures (positive, negative, and neutral). We used instructions to manipulate participants’ emotional responses to pictures according to emotion regulation strategic cues. By using two different forms of explicit test (recognition and free recall), we teased apart different influences of emotion regulation strategies on memory. Importantly, we found that cognitive reappraisal (cognitive up-regulation and cognitive down-regulation) enhanced two kinds of explicit memory performance relative to passive viewing and expressive suppression. Cognitive reappraisal was associated with enhanced explicit memory of the emotional event. There were no significant differences in recognition performance or free recall performance between the passive viewing group and expressive suppression group. In addition, there were also significant differences among the memory scores for different emotional pictures. Participants recognized positive pictures faster than negative pictures and neutral pictures. Furthermore, recall scores were better for negative pictures than positive pictures, and recall scores were higher for positive pictures than neutral pictures.

Cognitive up-regulation significantly enhanced the emotional experience of positive, negative and neutral images, and cognitive down-regulation significantly reduced the emotional experience of emotional pictures. Compared with the passive viewing condition, expressive suppression reduced the pleasantness of positive images and neutral pictures, and increased the pleasantness of negative images. Participants effectively changed the emotional experience of emotional pictures after employing different emotion regulation strategies.

Cognitive up-regulation significantly enhanced the arousal from three emotional pictures, while cognitive down-regulation significantly reduced the arousal from the pictures. Self-reported arousal was lower in the expressive suppression condition than the passive viewing condition for positive images and neutral images, but not for negative images. Previous studies have shown that expressive suppression could increase negative emotions and reduce positive emotions (Brans et al., 2013). This shows that our results are reliable. According to the self-reported valence and arousal ratings, participants adopted these emotion regulation strategies to regulate their emotional experience of emotional pictures effectively.

Consistent with previous studies, cognitive reappraisal (including both up-regulation and down-regulation) reinforced memory more than passive viewing (Dillon et al., 2007; Sheppes and Meiran, 2007, 2008; Hayes et al., 2010). Importantly, we also found that cognitive reappraisal not only enhanced memory for negative pictures, but also for positive ones. In our opinion, during cognitive reappraisal, participants employed much of their cognitive resources to re-explain and refine the emotional material, producing a deeper cognitive analysis of the stimuli and leading to memory enhancement. In the classic model of limited-resource attention, attentional resources for cognitive processing are limited. When being assigned multiple tasks simultaneously, if a task occupied a large amount of resources, the other tasks could only use the remaining few (Ellis, 1985). In this study, participants only needed to code the pictures in the passive viewing. Whereas, under the experimental conditions of the emotion regulation strategies, participants had to complete two tasks simultaneously: (1) code and recall the picture, and (2) emotion regulation. While the two tasks both required attention resources, participants used distinct methods to regulate different emotions during cognitive reappraisal and expressive suppression. As a result, attention resources were distributed differently in these two regulation strategies (cognitive reappraisal and expressive suppression), resulting in a differential impact on explicit memory. During cognitive reappraisal, participants accomplished emotion regulation through re-evaluating pictures. Participants were asked to imagine either playing a central role in the picture’s scenario or, alternatively, engaging from a stranger’s perspective in order to change the relationship between the participant and the emotional stimulation (Dillon et al., 2007). For example, to increase the emotional experience, when viewing a picture of a burning man, participants in the cognitive up-regulation group were asked to imagine him or herself or a loved one experiencing the burning depicted. On the other hand, participants in the cognitive down-regulation group were asked to view the image as objectively as possible to distance them from the scenario, thus lowering the emotional experience. These behaviors are also a type of coding and deep processing. Therefore, cognitive reappraisal employed a deeper level of processing, which enhanced the memory.

Neuroimaging findings also support the conclusion that cognitive reappraisal enhanced memory by deep processing of the picture. When cognitive reappraisal has been used to up-regulate or down-regulate emotional responses, researchers have found an increase in brain activity in the regions related to cognitive control (Ochsner et al., 2004; McRae et al., 2010) and cognitive processing (Dobbins and Wagner, 2005) was observed. Using neuroimaging techniques, Hayes et al. (2010) have found that the left inferior frontal gyrus and the hippocampus showed greater co-activation in this process relative to a passive viewing condition. Both of these regions are related to deep coding. On the other hand, compared with passive viewing, cognitive reappraisal reduced brain activity in the amygdala which was associated with negative emotion (Ochsner et al., 2004). These results have shown that cognitive reappraisal could enhance explicit memory of emotional stimuli, despite the suppression of arousing emotions. This indicates that cognitive reappraisal enhances memory by producing more in-depth cognitive processing.

Knight and Ponzio (2013) have shown that up-regulation of emotion helped with memory, while down-regulation of emotion led to impaired performance of free recall of pictures. We speculated that, in their study, the discrepancy between the up-regulation and down-regulation groups was related to how effectively the emotions were regulated; that is, more successful emotion regulation resulted in better memory performance. Evaluation of the self-reported arousal in Knight and Ponzio’s study showed that the decrease from the passive viewing condition to the down-regulation condition (0.51) was much less than the increase gained from up-regulation (1.16). In contrast, in this present study, the levels of change for the down-regulation (1.09) and up-regulation (0.82) were comparable. Knight and Ponzio’s study did not reveal that down-regulation of emotion enhance the memory scores of free recall. We suspected the reason maybe was that participants in Knight and Ponzio’s study failed to manage their emotions by using the down-regulation of emotion regulation strategy and thereby failed to refine the emotional material. Therefore, the benefits of down-regulation on the free recall of emotional pictures might had gone undetected because emotions were not effectively regulated in Knight and Ponzio’s study.

The present study also demonstrated that, compared with passive viewing, expressive suppression significantly decreased memory performance for the positive pictures. On the other hand, no significant difference was observed for the negative and neutral groups. The decreased memory performance for the positive picture group was due to memory impairment caused by the expressive suppression strategy. This damage occurred since monitoring and adjusting facial expressions largely utilized cognitive resources, thus leaving fewer of these for picture coding. However, when compared with passive viewing, emotion regulation strategies based on expressive suppression did not reduce the recognition score for negative images. We speculated that this is because expressive suppression only successfully decreased arousal of positive emotions, and did not regulate self-reported arousal of negative pictures effectively. The arousal ratings were 6.26 for passive viewing and 6.52 for expressive suppression, which was not a significant difference. In previous studies showing that expressive suppression reduced memory test scores from emotional stimuli, expressive suppression also significantly reduced arousal ratings for emotional stimuli compared with passive viewing (Dillon et al., 2007; Hayes et al., 2010). Thus, in these studies, by controlling expression of emotional behavior, expressive suppression succeeded in achieving the purpose of emotion regulation. Since attention resources were occupied by the act of controlling expression, cognitive resources for memorizing emotional material were diminished, and as such the memory performance from emotional material was compromised.

As two types of explicit memory test, recognition and free recall are two paradigms for measuring recollection and familiarity. Recollection is the extraction of specific information from previously learned projects, while familiarity evaluates the overall similarity between previously learned projects and test projects. Familiarity is the feeling of “déjà vu” when there is insufficient source information (Yonelinas, 2002; Liu et al., 2015). We therefore believe that, in recognition tasks, global processing is relied upon more; while in free recall tasks, the extraction of local information has greater importance.

Fredrickson proposed a broaden-and-build theory, suggesting that positive emotion could broaden an individual’s momentary thought–action repertoire, including the expansion of attention, cognition, action and other variables (Fredrickson and Levenson, 1998). Fredrickson suggested that both negative and positive emotions were adaptive. Negative emotion can make a person benefit from a threatening situation, since it will result in specific action tendencies and narrows a person’s thought-action resources in life-threatening situations, so that the individual will focus more on the momentary situation, and makes a quick, decisive action to survive. Fredrickson employed a global-local visual processing task (Fredrickson and Branigan, 2005), and found that participants in a positive emotional state were more likely to choose a figure that was consistent with the global pattern of the standard figure, indicating that positive emotions can broaden attention scope and produce a wider focus on global rather than local characteristics. Whereas individuals in a positive emotional state have a wider attention span and are more inclined to adopt global processing, participants in a negative emotional state have a narrowed attention span and are more inclined to use local processing. Therefore, in the recognition task, which relies on global judgment, the memory performance with positive pictures was better than with negative pictures. Conversely, free recall task, which relies on the memory of local information, results with better performance for negative pictures.

In summary, this study demonstrated that both cognitive up-regulation and down-regulation strategies improved the recognition of emotional information and free recall test scores. We speculated that cognitive reappraisal influenced memory by affecting the degree of material processing. For instance, cognitive reappraisal enhanced memory by directing more attention to deeper processing of stimuli. On the other hand, since attention was diverted away from the stimuli during expressive suppression, memory performance was impaired. We conclude that cognitive reappraisal strategies not only effectively regulate our emotions, but also improve our memory of emotional events.

This study primarily investigated the effects of different emotion regulation strategies on explicit memory. In future studies, the effects of emotion regulation strategies on implicit memory could be investigated by using other methods such as perceptual identification. In the current study, measurements of memory were all performed in real-time. To investigate the lasting effects of regulation strategies on memory, in future studies memory performance can also be measured after a certain period.

## Ethics Statement

This study was carried out in accordance with the recommendations of ‘name of guidelines, name of committee’ with written informed consent from all subjects. All subjects gave written informed consent in accordance with the Declaration of Helsinki. The protocol was approved by the ‘East China Normal University of Committee on Human Research Protection.’

## Author Contributions

Concept and design of study: YW and JC. Data acquisition, analysis, and interpretation: YW and BH. Drafting the work or revising it critically for important intellectual content: YW and JC. Final approval of the version to be published: YW, JC, and BH. Agreement to be accountable for all aspects of the work in ensuring that questions related to the accuracy or integrity of any part of the work are appropriately investigated and resolved: YW, JC, and BH.

## Conflict of Interest Statement

The authors declare that the research was conducted in the absence of any commercial or financial relationships that could be construed as a potential conflict of interest.
